# Mafenide derivatives inhibit neuroinflammation in Alzheimer's disease by regulating pyroptosis

**DOI:** 10.1111/jcmm.16984

**Published:** 2021-10-10

**Authors:** Chenyang Han, Qiaohong Hu, Anqi Yu, Qingcai Jiao, Yi Yang

**Affiliations:** ^1^ State Key Laboratory of Pharmaceutical Biotechnology School of Life Science Nanjing University Nanjing China; ^2^ Department of Pharmacy The Second Affiliated Hospital of Jiaxing University China

**Keywords:** inflammatory response, neuroinflammation, pyroptosis, sulfonamide

## Abstract

The main mechanism of pyroptosis is Caspase‐1–mediated GSDMD cleavage, and GSDMD is also the executive protein of pyroptosis. Our previous study has shown that mafenide can inhibit pyroptosis by inhibiting the GSDMD‐Asp275 site to suppress cleavage. In this study, sulfonamide was used as the parent nucleus structure to synthesize sulfa‐4 and sulfa‐20. Screening of drug activity in the pyroptosis model of BV2 and iBMDM cell lines revealed the efficacy of five compounds were superior to mafenide, which exerted a better inhibitory effect on the occurrence of pyroptosis. For in vivo assay, Sulfa‐4 and Sulfa‐22 were intervened in the neuroinflammation APP/PS1 mice. As a result, the administration of Sulfa‐4 and Sulfa‐22 could significantly inhibit the activation of microglia, decrease the expression of inflammatory factors in the central nervous system and simultaneously suppress the production of p30‐GSDMD as well as the expression of upstream NLRP3 inflammasome and Caspase‐1 protein. Immunoprecipitation and Biotin‐labelled assay confirmed the targeted binding relationship of Sulfa‐4 and Sulfa‐22 with GSDMD protein in the iBMDM model in vitro. In this study, we investigated a new type inhibitor of GSDMD cleavage, which exerted a good inhibitory effect on pyroptosis and provided new references for the development of inflammatory drugs in the future.

## 1 BACKGROUND

Pyroptosis is a type of programmed death that depends on inflammatory. Intracellular inflammasomes through the classical pyroptosis pathway of Caspase‐1 or the non‐classical pathway of Caspase‐4/5/11 to further cleavage the executive protein GSDMD to form p30‐GSDMD to open the cell membrane pores.^1^, ^2^ Meanwhile, the inflammasome promotes the maturation, which are released through membrane pores.^3^ During the whole process of pyroptosis, inflammasome and GSDMD are key factors. GSDMD is a member of the Gasdermin protein family.^4^, ^5^ NLRP3 inflammasome can recruit ASC to form bodies, which is further served as a medium to cleave pro‐Caspase‐1.^6^, ^7^ GSDMD is formed under the cleavage of Caspase‐1. After GSDMD cleavage, p30‐GSDMD is formed. After p30 oligomerization, it can open the membrane pore.^8^ GSDMD cleavage is a critical step in the occurrence of pyroptosis. In Alzheimer's disease (AD), β‐amyloid deposition can activate NLRP1 inflammasome, mediate the occurrence of pyroptosis and trigger local inflammation.^9^, ^10^

The cleavage of GSDMD is mainly formed at Asp275‐276. Previous studies have already demonstrated that mafenide can inhibit the cleavage of GSDMD by targeting GSDM‐Asp275, thereby inhibiting the occurrence of pyroptosis. Virtual docking further identified that the sulfonamide parent nucleus of the mafenide had a good binding capacity with Asp275.^11^

## 2 MATERIALS AND METHODS

### 2.1 Design and synthesis methods of sulfonamide compounds

Show in Supplementary material.

### 2.2 Induction of pyroptosis and drug intervention methods

iBMDM (Procell, China WUHAN) and BV2 (Procell, China WUHAN) were obtained. iBMDM were cultured in 10% supercalf serum with 1% antibiotic/antimycotic and puromycin (3 μg/ml). BV2 were cultured in MEM containing 10% FBS and 1% antibiotic/antimycotic.

The pyroptosis of iBMDM and BV2 cells was induced by a combination of lipopolysaccharide (LPS) (1 μg/ml) and Nigericin (10 μM). iBMDM and BV2 cells of the logarithmic phase were divided into two groups: one group was intervened with small molecule compounds for 2 h, and the control group was added with DMSO. After changing into the serum‐free medium, cells were induced by LPS (1 μg/ml) for 4 h, followed by pyroptosis induction by Nigericin (10 μM) for 2 h. After cell intervention, cells and culture medium were collected, followed by detection of the expression of IL‐1β and TNF‐α by ELISA to calculate IC50.

Detection of the pyroptosis‐inhibiting role and mechanism of Sulfa‐4 and Sulfa‐22 small molecules in BV2 cells:

1. Lactate dehydrogenase (LDH) assay: cells were incubated with Nigericin for 2 h to induce pyroptosis, performed LDH release rate detection according to the operating instructions of the kit (results were shown as %).
2. PI absorption rate: After Nigericin intervention within 2 h, the PI absorption rate was measured every 10 min. Cells were treated with 1 μg/ml PI, 120 nM NaCl, 5 mM Glucose, 1.5 mM CaCl_2_, 1 mM magnesium chloride and 0.1% bovine serum albumin (BSA). Cells were tested for absorbance at 533/617 nm. PI absorption rate (%) = (OD_Sample_ − OD_background_)/(OD_maximum_ − OD_background_).
3. PI, Hoechst 33258 staining: Cells were subjected to staining after Nigericin intervention for 2 h. For Hoechst 33258 staining, cells were incubated with Hoechst 33258 staining solution (Beyotime Biotechnology Co., Ltd.) for 15 min and washed with PBS. For PI staining, cells were incubated with PI staining reagent (1 μg/ml) for 30 min and washed with PBS. Observed the number of positive cells, calculated the number of positive cells in each field for statistics
4. Western blot: BV2 cells were treated with Nigericin for 2 h, collected, washed twice with PBS and lysed with pre‐cool 1.0 ml RIPA lysis buffer (Beyotime Biotechnology Co., Ltd.) on ice for 30 min. The protein sample was added with 5× loading buffer to a final volume of 20 μl with and boiled for 8 min. The protein samples were subsequently subjected to electrophoresis at 80V voltage and further at 120V. The PVDF membrane was blocked with 5% skimmed milk powder for 2h, incubated with primary monoclonal antibody diluted with TBST, washed with TBST twice and incubated with HRP‐labelled goat anti‐rabbit secondary antibody (Abcam), followed by ECL for visualization. The results were expressed as the comparison of the optical density between the target protein and the internal control. The monoclonal antibody dilution ratio of GSDMD and p30‐GSDMD (key pyroptosis protein) was 1:500 (Abcam); the monoclonal antibody dilution ratio of NLRP3 and Caspase‐1(key protein of NLRP3 inflammasome) was 1:800 (Abcam); the dilution of HRP‐labelled IgG antibody was 1:1000 (Abcam).
5. Detection of cytokine expression by ELISA: BV2 cells were treated with Nigericin for 2 h, and the supernatant was thereafter collected every 30 min. The supernatant was subjected to the detection of inflammatory factors taken for the detection of inflammatory factors. ELISA kits for IL‐1β, IL‐18 and TNF‐α (Nanjing Jiancheng Biotechnology Co., Ltd.) were used for detection according to the manufacturer's instruction. The absorbance value was measured at 450nm by microplate reader (BioTek), and the results were expressed as pg/ml.
6. Immunofluorescence staining: The coverslip was placed in a 6‐well plate for IF staining. In brief, cells were fixed with freshly prepared 4% paraformaldehyde (PFA) for 10 min, wash with PBS, permeabilized with 0.2% Triton X‐100, blocked them with 2% BSA, incubated with monoclonal antibody against GSDMD and p30‐GSDMD (Abcam) (dilution 1:300) at room temperature for 1 h, wash with PBS for three times, reacted with IgG antibody for labelling, subjected to 0.5 μg/ml DAPI staining reagent (Solarbio) for nuclear staining, washed twice with PBS, mounted and observed under fluorescence microscope. The expression of GSDMD protein was detected in both cytoplasm and cell membrane, while p30‐GSDMD was mainly expressed on cell membrane.

### 2.3 The targeted binding relationship of Sulfa‐4 and Sulfa‐22 small molecules with GSDMD

Co‐immunoprecipitation (Co‐IP) assay: BV2 cells were inoculated into a 6‐well plate and separately treated with DMSO, LPS+Nigericin and Sulfa‐4 or Sulfa‐22 for 4 h. After washing with PBS, cells were added with 120 μl lysis buffer on ice, followed by collection of cells into EP tube. After centrifugation, the supernatant was transferred to a new EP tube and subjected to BCA assay to detect protein concentration. The sample was added with anti‐GSDMD monoclonal antibody (Abcam) (200 μg antibody/2μl sample) at 4°C overnight and reacted with protein G agarose affinity matrix in a shaker at 4°C for 4 h. The supernatant was collected and washed with pre‐cooled PBS for four times, followed by detection of GSDMD by WB.

### 2.4 Intervention effects of Sulfa‐4 and Sulfa‐22 small molecules on APP/PS1 mice

#### 2.4.1 Intervention of Sulfa‐4 and Sulfa‐22 on APP/PS1 mice

Thirty 4‐month‐old APP/PS1 mice were used for the experiment. Mice in the Control group were conventionally raised, followed by gastric administration of 5 mg/kg of Sulfa‐4 or Sulfa‐22. After 14 days, mice were sacrificed by CO_2_. The cerebrospinal fluid (CSF) and peripheral blood from the tail vein of mice were collected and centrifuged, followed by the storage of the supernatant. After all samples were obtained, ELISA kits were used to detect the expression of inflammatory factors IL‐1β, IL‐18 and TNF‐α. The cortex, hippocampus and striatum tissue of mice were extracted for protein sample, followed by detection of the expression levels of GSDMD and p30‐GSDMD by WB.

#### 2.4.2 IBA‐1 and CD11c by IF assay

The brain tissue of mice was fixed with 4% FPA. The brain tissue was dehydrated with 15%–30% sucrose solutions. The slices were washed with PBS, incubated with monoclonal antibodies against CD11c (dilution 1:50) and IBA‐1 (dilution 1:50) (Abcam) at 4°C overnight, incubated with fluorescent antibody (d ilution1:50) in the dark for 1 h, washed with PBS for three times and mounted with anti‐fluorescence quencher, followed by observation under microscope. Image‐Pro Plus was used to count the number of positive cells.

### 2.5 Statistical analysis

Measurement data were shown as $\bar{x}$±s. SPSS 22.0 was used for data analysis and processing. Two independent sample t test analysis was used for comparison between two groups; LSD method was used for the subsequent comparisons between the two groups. One‐way ANOVA was used for comparison among three groups and above, and all the above‐mentioned tests were two‐sided, and *p *< 0.05 indicated statistical significance.

## 3 RESULTS

### 3.1 The effect and mechanism of Sulfa‐4 and Sulfa‐22 on pyroptosis in BV2 cells

**FIGURE 1:**
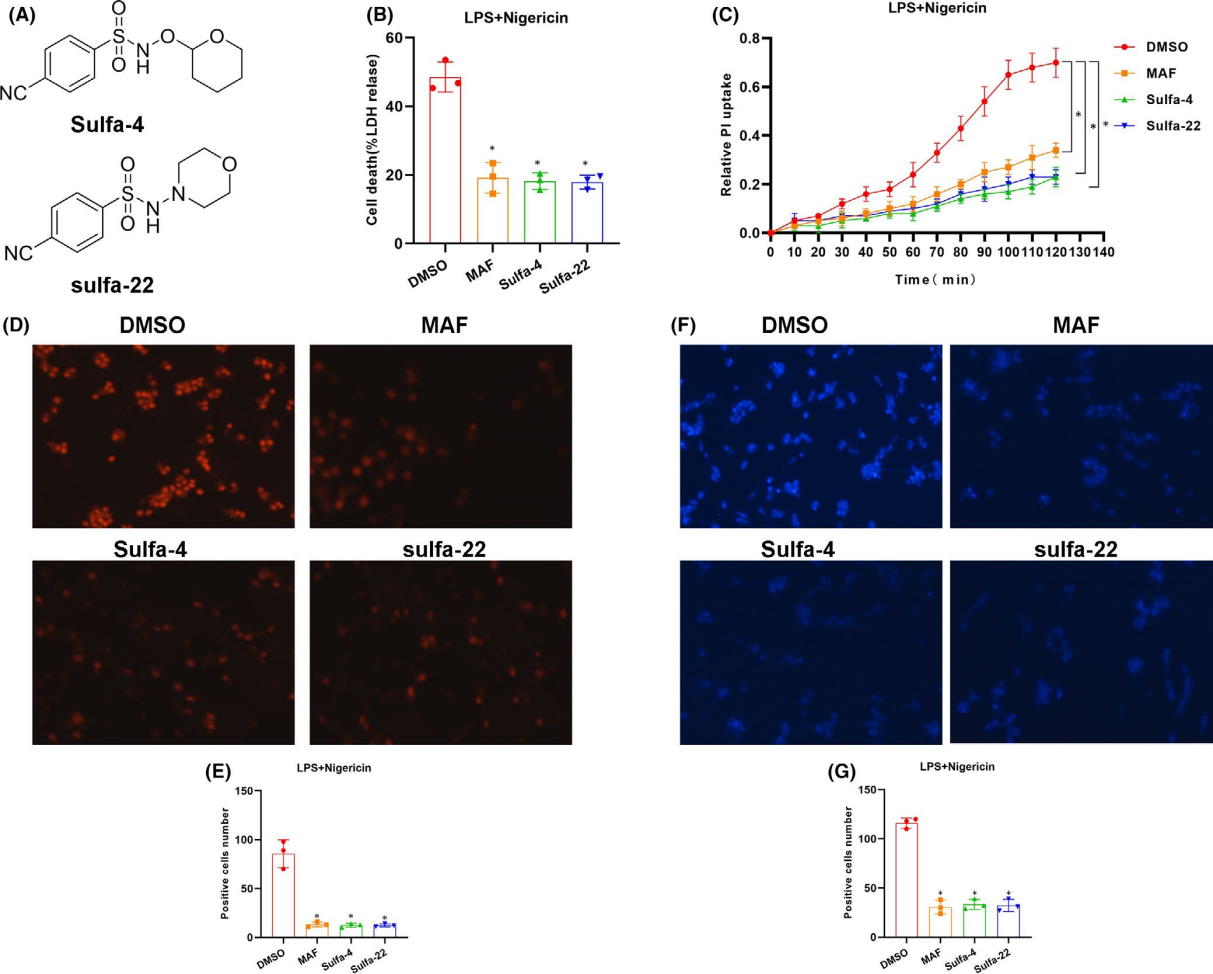
Sulfa4/22 on pyroptosis in BV2 cells. (A) The structure of Sulfa‐4 and Sulfa‐22 compounds. (B) Results of LDH release rate (n = 5): The LDH release rate was relatively high in the DMSO group, which was down‐regulated in the MAF and Sulfa‐4/22 groups decreased. Comparison with the DMSO group, *p<0.05. (C) of PI uptake rate (n = 3): The PI uptake rate was relatively high in the DMSO group, which was decreased in the MAF and Sulfa‐4/22 groups. Comparison with the DMSO group, *p < 0.05. (D‐E) PI staining (n = 5): Positive cells were significantly higher in the DMSO group than that of MAF and Sulfa‐4/22 groups. Comparison with DMSO group, *p < 0.05. (F‐G) Hoechst 33258 staining (n = 5): Positive cells was significantly higher in the DMSO group than that of MAF and Sulfa‐4/22 groups. Comparison with DMSO group, *p < 0.05

**FIGURE 2:**
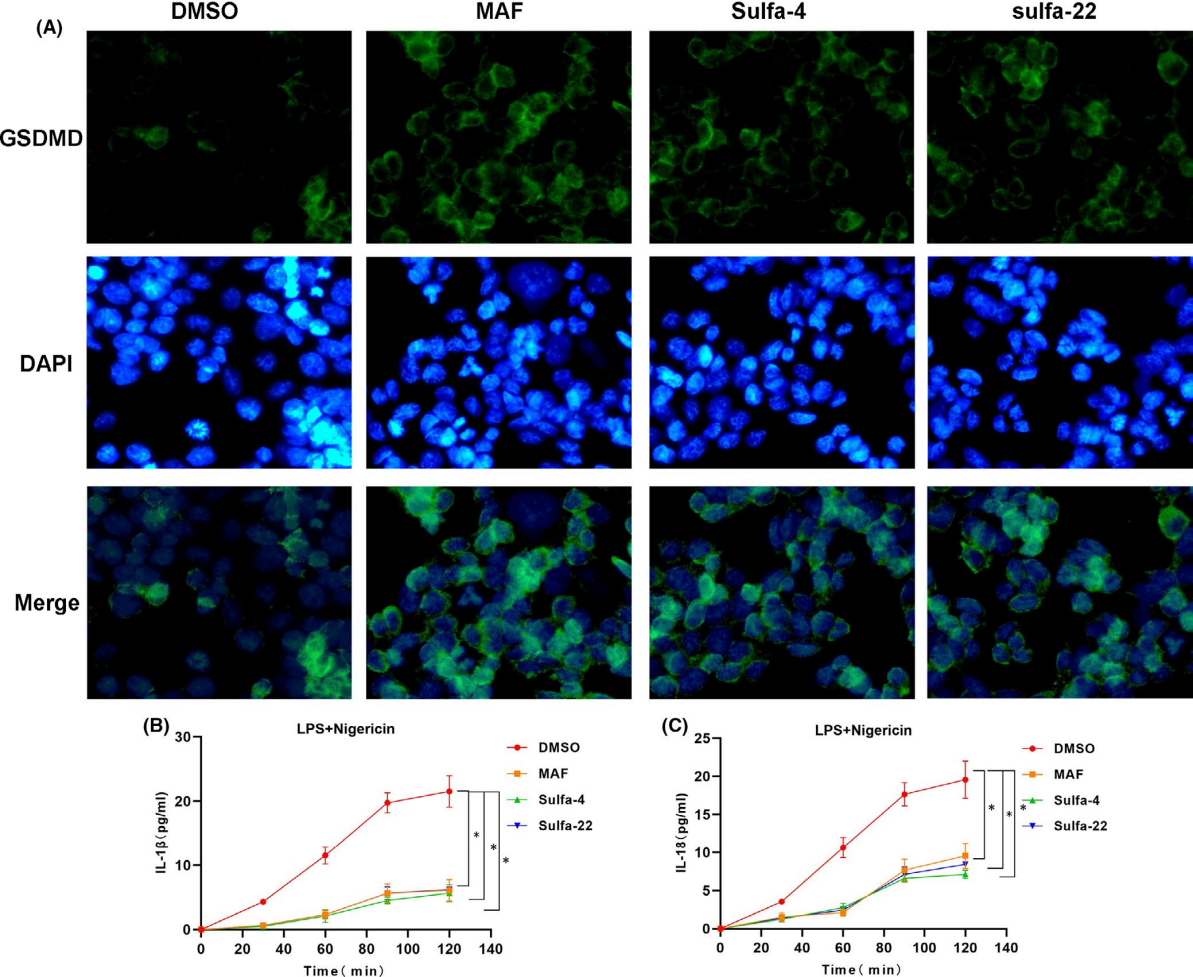
Sulfa4/22 on the release of GSDMD and inflammatory factors. (A) Results of GSDMD IF staining (n = 5): The expression level of GSDMD was relatively lower in the DMSO group, indicating the cleavage of GSDMD. While the expression of GSDMD protein was significantly up‐regulated in the MAF and Sulfa‐4/22 groups, suggesting the suppressed cleavage. (B‐C) Results of IL‐1β and IL‐18 release (n = 3): The release of inflammatory factors IL‐1β and IL‐18 was up‐regulated in the DMSO group, which was down‐regulated in the MAF and Sulfa‐4/22 groups. Comparison with the DMSO group, *p < 0.05

**FIGURE 3:**
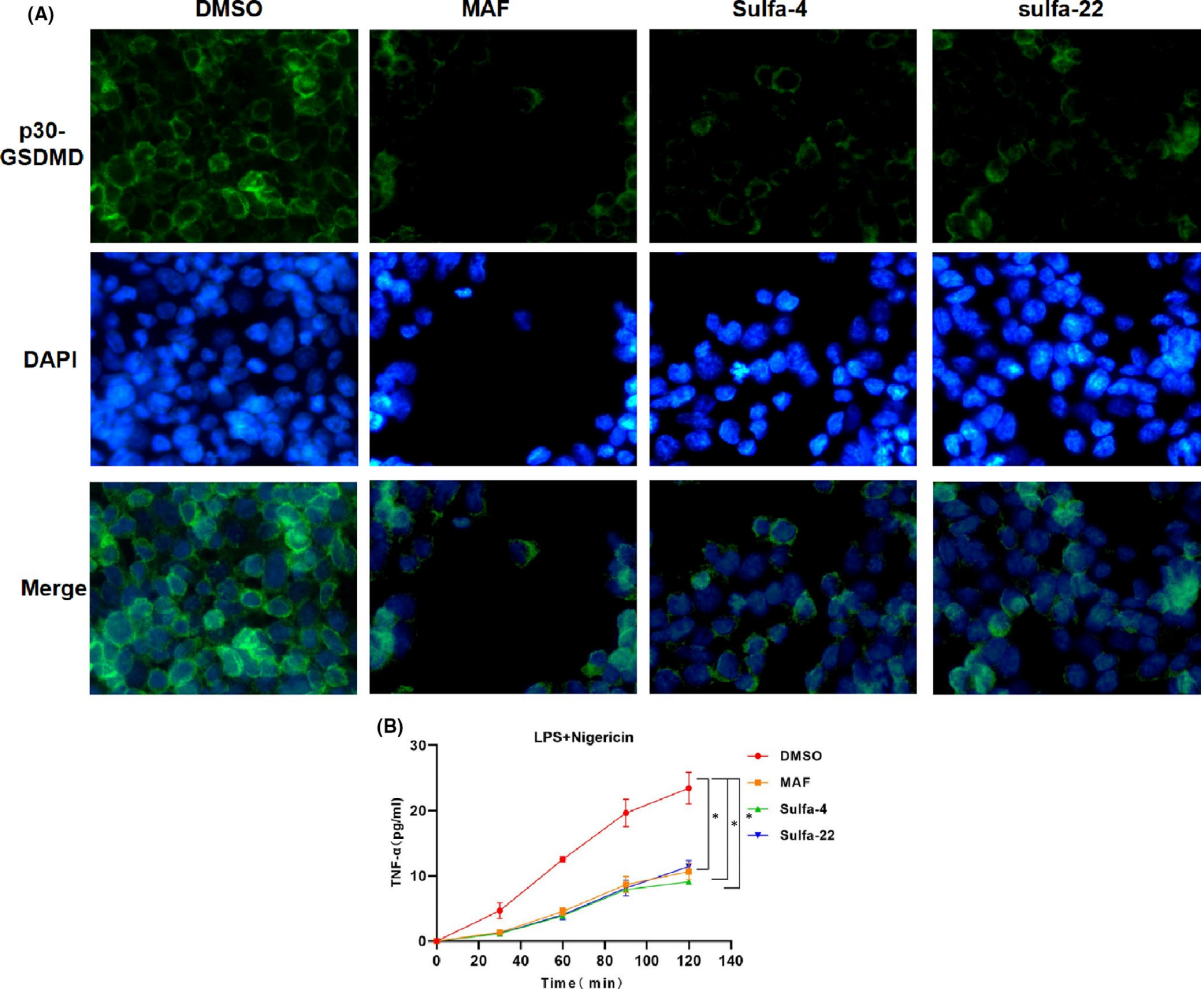
Sulfa4/22 on p30‐GSDMD and TNF‐α release. (A) p30‐GSDMD IF staining (n = 5): p30‐GSDMD was relatively high in the DMSO group, while p30‐GSDMD protein was significantly down‐regulated in the MAF and Sulfa‐4/22 groups, indicating the suppressed cleavage of GSDMD. (B‐C) Results of TNF‐α release (n = 3): The release of TNF‐αwas up‐regulated in the DMSO group, which was down‐regulated in the MAF and Sulfa‐4/22 groups. Comparison with the DMSO group, *p < 0.05

In this study, Sulfa‐4 (IC50 of 3μM) and Sulfa‐22 (IC50 of 5μM) were used for pretreatment in BV2 cells (Figure 1A), and 15μM mafenide (MAF) was used as the positive control. LDH release rate assay showed obvious LDH release in cells of the DMSO group, which was significantly down‐regulated in MAF group (positive control). In addition, the LDH release rate was down‐regulated in BV2 cells after Sulfa‐4/22 small molecule treatment (Figure 1B). PI uptake rate assay revealed that the PI uptake rate was significantly up‐regulated in the DMSO group, while the uptake rate in the MAF group was significantly down‐regulated; meanwhile, Sulfa‐4/22 small molecules could also down‐regulate the PI uptake rate in BV2 cells (Figure 1C). Moreover, PI staining and Hoechst 33258 staining showed that the number of positive cells was relatively more in the DMSO group, which was significantly down‐regulated in MAF and Sulfa‐4/22 groups compared to the DMSO group (*p *< 0.05) (Figure 1D‐G). IF staining showed that the fluorescence intensity of GSDMD in the DMSO group was relatively low, which was significantly lower than that of the MAF and Sulfa‐4/22 groups, indicating that GSDMD was cleaved in the DMSO group. In addition, p30‐GSDMD had stronger fluorescence intensity in the DMSO group, which was significantly higher than that of MAF and Sulfa‐4/22groups, and p30‐GSDMD was mainly expressed on the cell membrane (Figures 2A, 3A). Inflammatory factor release assay showed that the expression levels of inflammatory factors IL‐18, IL‐1β and TNF‐α in the DMSO group were increased over time, indicating that the opening of cell membrane pores promoted the release of inflammatory factors. However, the expression level of inflammatory factors was significantly lower in the MAF and Sulfa‐4/22 groups than that of the DMSO group (Figures 2B,C, 3B). Taken together, Sulfa‐4 and Sulfa‐22 could inhibit the cleavage of GSDMD to play an anti‐pyroptosis effect.

### 3.2 Sulfa‐4/22 on inhibiting pyroptosis in BV2 cell and the validation of targeting GSDMD

**FIGURE 4:**
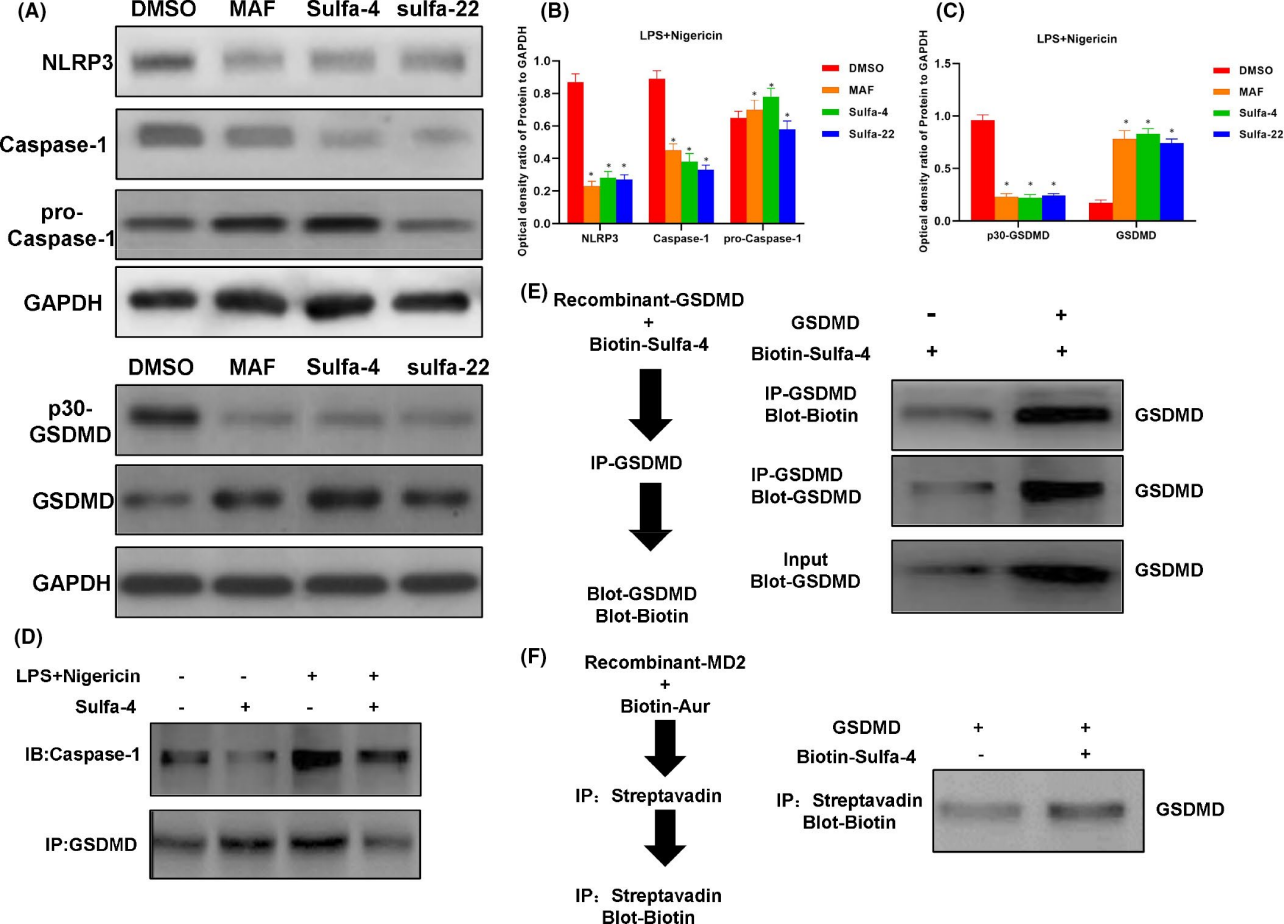
Sulfa‐4/22 in inhibit pyroptosis in BV2 cells, and the binding validation with GSDMD. (A‐C) Effects of Sulfa‐4/22 on the expression of key pyroptosis proteins (n = 3): NLRP3 and Caspase‐1 were relatively high in the DMSO group. Sulfa‐4/22 could inhibit NLRP3 and Caspase‐1. In the DMSO group, GSDMD was relatively high, and p30‐GSDMD was relatively low. Comparison with the DMSO group, *p < 0.05. (D) Validation of the binding between GSDMD and Sulfa‐4 by Co‐IP: Sulfa‐4 could directly bind to GSDMD, which had no binding relationship with Caspase‐1. (E‐F) Validation of the binding between GSDMD and Sulfa‐4 by pull‐down assay: Sulfa‐4 could directly bind to GSDMD

**FIGURE 5:**
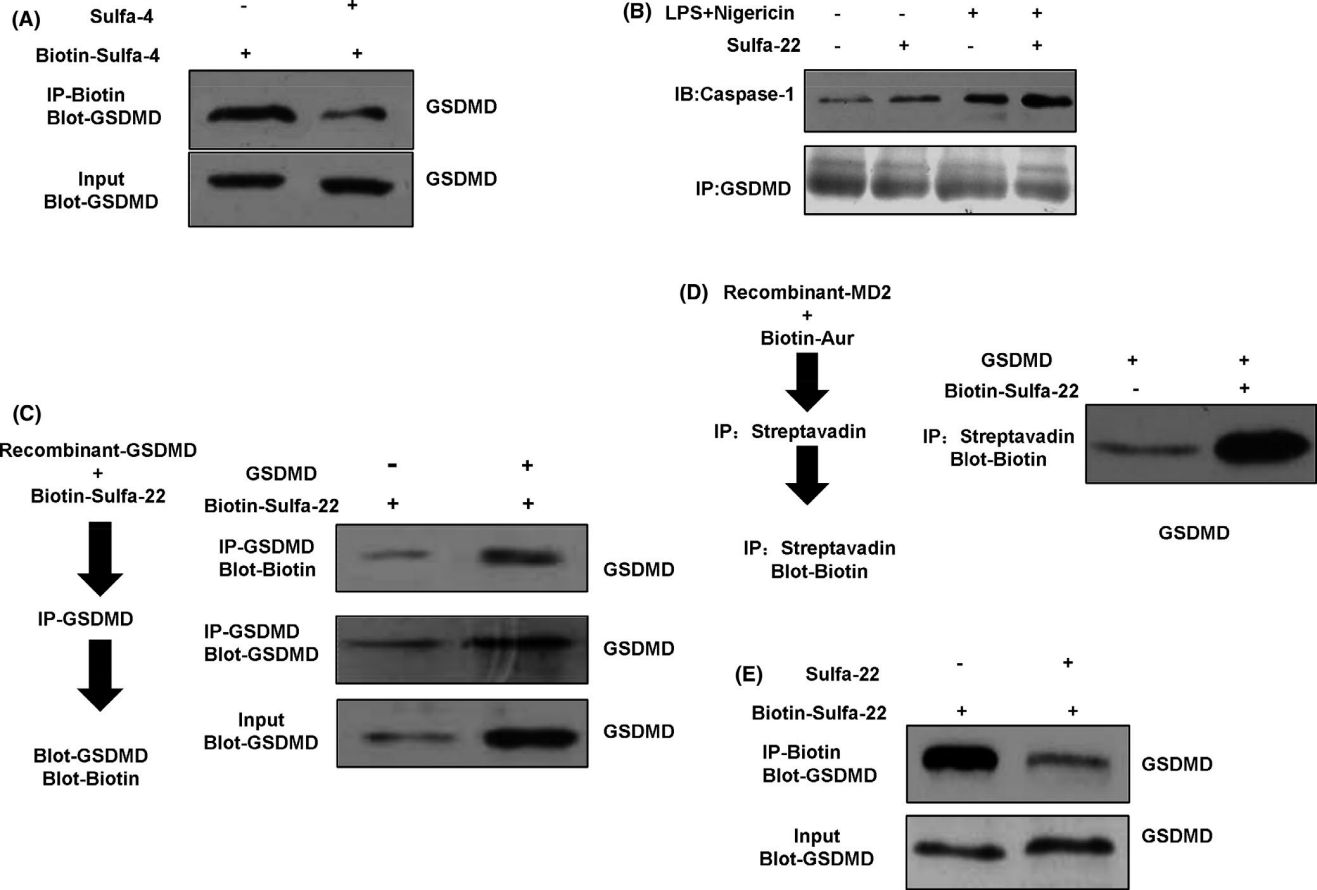
Validation of the targeted binding relationship between Sulfa4/22 and GSDMD. (A) Competitive assay showed that the Biotin‐labelled Sulfa‐4 could be replaced by the unlabelled Sulfa‐4, indicating that the two sites were both GSDMD. (B) Validation of the binding between GSDMD and Sulfa‐22 by Co‐IP: Sulfa‐22 could directly bind to GSDMD, which had no binding with Caspase‐1. (C‐D) Validation of the binding between GSDMD and Sulfa‐22 by pull‐down assay: Sulfa‐22 could directly bind with GSDMD. (E) Competitive assay revealed that Biotin‐labelled Sulfa‐22 could be replaced by unlabelled Sulfa‐22, indicating that the two sites were both GSDMD

The expression of NLRP3 and Caspase‐1 was up‐regulated in the DMSO group, while the expression of Pro‐Caspase‐1 was not significantly changed. The expression of NLRP3 and Caspase‐1 was down‐regulated in the MAF and Sulfa4/22 groups, which was consistent with the IF results. For the DMSO group, the expression of GSDMD was down‐regulated, while the expression of p30‐GSDMD was up‐regulated, indicating that GSDMD was cleaved. However, for the MAF and Sulfa4/22 groups, the cleavage of GSDMD was inhibited, and the expression of p30‐GSDMD was down‐regulated (Figure 4A‐C). Co‐IP assays revealed that Sulfa‐4 and Sulfa‐22 could directly interact with GSDMD instead of Caspase‐1. Caspase‐1 can bind to GSDMD and can cleave GSDMD to form p30‐GSDMD (Figures 4D, 5A‐B). The pull‐down assay on biotin‐labelled Sulfa‐4 and Sulfa‐22 showed that Sulfa‐4 and Sulfa‐22 could combined with GSDMD (Figures 4E, 5C‐D). Competitive assay on the unlabelled Sulfa‐4 and Sulfa‐22 showed that Sulfa‐4 and Sulfa‐22 could compete with Biotin‐labelled small molecules to bind to GSDMD, which also indicated the binding relationship of small molecules Sulfa‐4 and Sulfa‐22 with GSDMD (Figures 4F, 5E).

### 3.3 Effects of Sulfa‐4/22 on microglia and inflammatory factor release in APP/PS1 mice

**FIGURE 6:**
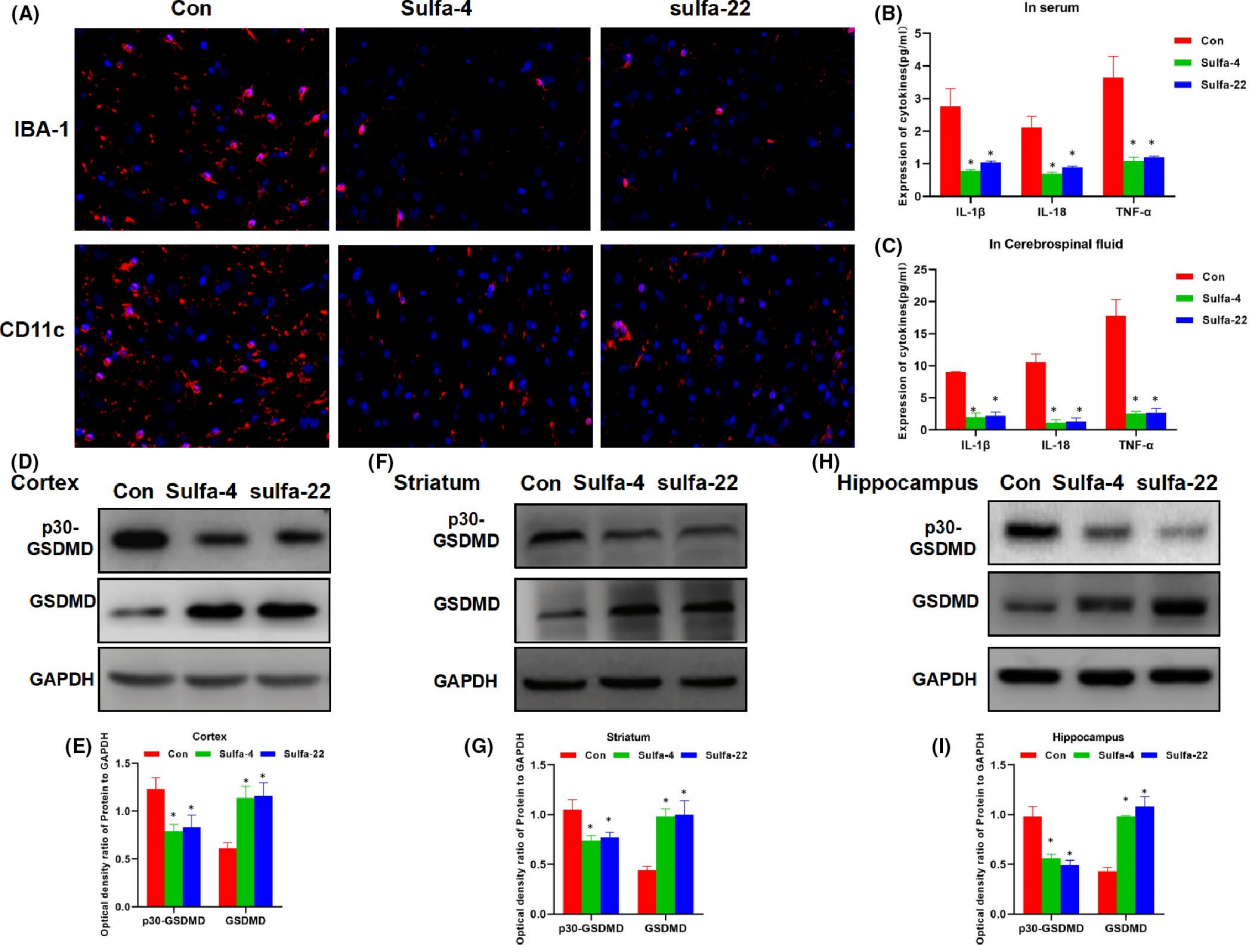
Effects of Sulfa4/22 on microglia activation and the release of inflammatory factors in APP/PS1 mice. (A) Detection of IBA‐1 and CD11c expression by IF staining: There was obvious activation of microglia, obvious expression of IBA‐1 and CD11c in the Con group. However, the expression of IBA‐1 and CD11c was significantly down‐regulated, and the activation of microglia was inhibited in Sulfa‐4 and Sulfa‐22 group. (B‐C) Expression of inflammatory factors in peripheral blood and CSF: The expression levels of inflammatory factors in peripheral blood and CSF were relatively high in Con group, which were significantly down‐regulated in Sulfa‐4 and Sulfa‐22 group. Comparison with Con group, *p < 0.05. (D‐I) Expression level of GSDMD and p30‐GSDMD in mouse cortex, hippocampus, and striatum: The expression GSDMD was relatively low, while p30‐GSDMD expression was relatively high in cortex, hippocampus and striatum. The cleavage of GSDMD was inhibited in Sulfa‐4 and Sulfa‐22 groups, GSDMD expression level was significantly up‐regulated, while the level of p30‐GSDMD was down‐regulated. Comparison with the Con group, *p < 0.05

APP/PS1 is a commonly used mouse model of AD, with obvious senile plaques and microglia activation at the age of 4 months. Therefore, Sulfa‐4 and Sulfa‐22 were used for intervention. As a result, after Sulfa‐4 and Sulfa‐22 intervention, the expression of microglia markers IBA‐1 and CD11c was significantly decreased, and the levels of inflammatory factors in peripheral blood and CSF were down‐regulated, indicating that Sulfa‐4 and Sulfa‐22 could attenuate inflammatory response and microglia activation (Figure 6A‐C). The expression of p30‐GSDMD in the cortex, hippocampus and striatum was down‐regulated, while the expression of GSDMD was up‐regulated, indicating the inhibitory cleavage of GSDMD (Figure 6D‐I).

## 4 DISCUSSION

Alzheimer's disease is a degenerative disease and the main cause of cognitive impairment.^12^ The pathological features of AD are mainly β‐amyloid protein deposition and Tau protein phosphorylation.^13^, ^14^ However, the exact pathological mechanism of AD has not yet been revealed. In recent years, many studies have found that neuroinflammation plays an important role in the occurrence and development of AD. In neuroinflammation, microglia play an important role and are also one of the main cells that release inflammatory factors.^15^, ^16^ Pyroptosis is a type of cell death, which involves the activation of Caspase‐1‐GSDMD. Caspase‐1 is the key protein for pro‐IL‐1β cleavage and maturation; therefore, pyrolysis is mostly mediated by Caspase‐1. However, it is GSDMD protein that definitely mediates the occurrence of pyroptosis.^7^ The level of GSDMD in normal cells is low, and GSDMD can be induced by LPS. The massive expression of Caspase‐1 can cleave GSDMD to form p30‐GSDMD protein, and the oligomerized p30‐GSDMD is located on the cell membrane to open the cell channel to promote the release of inflammation, which is considered as the main cause of pyroptosis.^17^ Previous studies have shown that inhibition of pyroptosis can inhibit the release of inflammatory factors. The key to inhibition of pyroptosis is in the inhibition of GSDMD protein, which theoretically can inhibit the cleavage of GSDMD. Our team has previously shown that MAF can target the GSDMD and inhibit the cleavage of GSDMD.

In this study, MAF was used as the lead compound, and we found that the sulfonamide structure was the pharmacodynamic structure of Asp275. Therefore, we tried to perform small molecule modification at the binding sites by skeleton migration, aiming to supplement the group to fill the binding cavity. In consideration of binding stability, the amino structure was converted to the cyano group, which could, on the one hand, increase the binding of π‐π bonds, and on the other hand, improve the fat solubility. In vitro assays revealed that Sulfa‐4 and Sulfa‐22 had better efficacy. The comparison of compound efficacy showed that by detecting inflammatory factors and GSDMD, we found that two molecules, Sulfa‐4/22 had better efficacy. To further verify its effect, we performed experiments in BV2 cells. Consequently, Sulfa4/22 could significantly inhibit the occurrence of pyroptosis, and the effect at low concentrations was similar to that of MAF. Sulfa4/22 could effectively inhibit inflammatory factors at low concentrations. Pull‐down assays and Co‐IP assays proved that Sulfa4/22 could bind with GSDMD instead of Caspase‐1. Because Caspase‐1 and GSDMD interact with each other, the results also demonstrate the target of Sulfa4/22. The IF staining clearly showed that Sulfa4/22 increased the expression of GSDMD and decreased the expression of p30‐GSDMD. Because p30‐GSDMD is cleaved from GSDMD, the up‐regulated expression GSDMD fully indicated that Sulfa4/22 inhibited the cleavage process. To further validate the role of Sulfa4/22 in neuroinflammation, APP/PS1 mouse model, a common AD model, was used for intervention.^18^ Sulfa4/22 intervention could decrease the level of inflammatory factors, especially in CSF. CSF communicates directly with the central nervous system and intuitively reflects the inflammation level of the central nervous system; therefore, detection of CSF could help to determine that MAF has an effect on neuroinflammation. More interestingly, Sulfa4/22 could inhibit microglia activation and decrease the expression of IBA‐1 and CD11c. In addition, the inhibited GSDMD cleavage is also detectable in the hippocampus and cortex. Collectively, it can be concluded that Sulfa4/22 can inhibit pyroptosis to play an anti‐inflammatory effect, and its mechanism is associated with the cleavage of GSDMD.

## 5 CONCLUSION

In this study, we designed Sulfa‐4 and Sulfa‐22 by using MAF as the lead compound. In vitro activity screening revealed that the pharmaceutical efficacy of two small molecules was superior to MAF. In vivo and in vitro assays also fully demonstrated that Sulfa4/22 could target the cleavage of GSDMD, inhibit the occurrence of pyroptosis and the release of inflammatory factors, exerting a certain effect on the neuroinflammation of AD.

## CONFLICT OF INTEREST

The authors declare that they have no conflict of interest.

## AUTHOR CONTRIBUTIONS

**chenyang han:** Conceptualization (equal); Data curation (equal); Investigation (equal); Resources (equal). **qiaohong hu:** Conceptualization (equal); Investigation (equal); Project administration (equal); Supervision (equal); Writing‐original draft (equal). **anqi yu:** Methodology (equal); Software (equal); Supervision (equal); Writing‐original draft (equal). **qingcai jiao:** Resources (equal); Validation (equal); Validation (equal); Writing‐original draft (equal); Writing‐original draft (equal); Writing‐review & editing (equal); Writing‐review & editing (equal). **yi yang:** Conceptualization (equal); Formal analysis (equal); Investigation (equal); Resources (equal).

## Supporting information

Supplementary material

## Data Availability

The data that support the findings of this study are available from the corresponding author upon reasonable request.

## References

[1] Cheng R , Feng Y , Zhang R , et al. The extent of pyroptosis varies in different stages of apical periodontitis. Biochim Biophys Acta Mol Basis Dis. 2018;1864(1):226‐235. 29066283 10.1016/j.bbadis.2017.10.025

[2] Wang Y , Shi P , Chen Q , et al. Mitochondrial ROS promote macrophage pyroptosis by inducing GSDMD oxidation. J Mol Cell Biol. 2019;11(12):1069‐1082. 30860577 10.1093/jmcb/mjz020 PMC6934151

[3] Bergsbaken T , Fink SL , Cookson BT . Pyroptosis: host cell death and inflammation. Nat Rev Microbiol. 2009;7(2):99‐109. 19148178 10.1038/nrmicro2070 PMC2910423

[4] Aglietti RA , Estevez A , Gupta A , et al. GsdmD p30 elicited by caspase‐11 during pyroptosis forms pores in membranes. Proc Natl Acad Sci. 2016;113(28):7858‐7863. 27339137 10.1073/pnas.1607769113 PMC4948338

[5] Sborgi L , Sebastian R , Mulvihill E , et al. GSDMD membrane pore formation constitutes the mechanism of pyroptotic cell death. EMBO J. 2016;35(16):1766‐1778. 27418190 10.15252/embj.201694696 PMC5010048

[6] Dong W , Zhu Q , Yang B , et al. Polychlorinated biphenyl quinone induces caspase 1‐mediated pyroptosis through the induction of pro‐inflammatory HMGB1‐TLR4‐NLRP3‐GSDMD signal axis. Chem Res Toxicol. 2019;32(6):1051‐1057. 30977640 10.1021/acs.chemrestox.8b00376

[7] Liu Z , Gan L , Xu Y , et al. Melatonin alleviates inflammasome‐induced pyroptosis through inhibiting NF‐κB/GSDMD signal in mice adipose tissue. J Pineal Res. 2017;63(1):e12414. 10.1111/jpi.12414 28398673

[8] Schneider KS , Groß CJ , Dreier RF , et al. The Inflammasome Drives GSDMD‐Independent Secondary Pyroptosis and IL‐1 Release in the Absence of Caspase‐1 Protease Activity. Cell Rep. 2017;21(13):3846‐3859. 29281832 10.1016/j.celrep.2017.12.018 PMC5750195

[9] Tan MS , Tan L , Jiang T , et al. Amyloid‐β induces NLRP1‐dependent neuronal pyroptosis in models of Alzheimer’s disease. Cell Death Dis. 2014;5(8):e1382. 25144717 10.1038/cddis.2014.348 PMC4454321

[10] Olsen I , Singhrao SK . Inflammasome involvement in Alzheimer’s disease. J Alzheimers Dis. 2016;54(1):45‐53. 27314526 10.3233/JAD-160197

[11] Han CY , Yang Y , Yu AQ , et al. Investigation on the mechanism of mafenide in inhibiting pyroptosis and the release of inflammatory factors. Eur J Pharmaceut Sci. 2020;147:105303. 10.1016/j.ejps.2020.105303 32173407

[12] Cummings J . Lessons learned from alzheimer disease: clinical trials with negative outcomes. Clin Transl Sci. 2018;11(2):147‐152. 28767185 10.1111/cts.12491 PMC5866992

[13] Lu D , Popuri K , Ding GW , et al. Multimodal and Multiscale Deep Neural Networks for the Early Diagnosis of Alzheimer's Disease using structural MR and FDG‐PET images. Sci Rep. 2018;8(1):5697. 29632364 10.1038/s41598-018-22871-z PMC5890270

[14] Joseph JT . Is Alzheimer disease a disease? Canadian J Neurol Sci/J Canadien Sci Neurol. 2019;46(s2):S60.

[15] Schetters STT , Gomez‐Nicola D , Garcia‐Vallejo JJ , et al. Neuroinflammation: microglia and T cells get ready to tango. Front Immunol. 2018;8:1905‐1912. 29422891 10.3389/fimmu.2017.01905 PMC5788906

[16] Song FJ , Zeng KW , Chen JF , et al. Extract of fructus schisandrae chinensis inhibits neuroinflammation mediator production from microglia via NF‐κ B and MAPK pathways. Chin J Integr Med. 2018;25(2):131‐138. 29790065 10.1007/s11655-018-3001-7

[17] Gao J , Qiu X , Xi G , et al. Downregulation of GSDMD attenuates tumor proliferation via the intrinsic mitochondrial apoptotic pathway and inhibition of EGFR/Akt signaling and predicts a good prognosis in non? Small cell lung cancer. Oncology Rep. 2018;40(4):1971‐1984. 10.3892/or.2018.6634 PMC6111570 30106450

[18] Xing XN , Sha S , Chen XH , et al. Active Immunization with DNA Vaccine Reduced Cerebral Inflammation and Improved Cognitive Ability in APP/PS1 Transgenic Mice by In Vivo Electroporation. Neurochem Res. 2015;40(5):1032‐1041. 25868754 10.1007/s11064-015-1559-4

