# Supplementary material for: Mafenide derivatives inhibit neuroinflammation in Alzheimer's disease by regulating pyroptosis
Source: J Cell Mol Med. 2021 Oct 10;25(22):10534–42. doi: 10.1111/jcmm.16984 (PMC8581306; doi:10.1111/jcmm.16984)
Supplement: Supplementary file 1 — Supplementary material [file JCMM-25-10534-s001.docx]

Design and synthesis methods of sulfonamide compounds

Asp275 site of GSDMD protein is the key site of Caspase-1 cleavage. The docking between MAF and GSDMD protein showed that Docking score was -5.720, with formed interactions; hydrogen bonds were SER30, ARG53, TYR54, CYS56; hydrophobic interactions included PHE34, TYR54, CYS56, VAL57, VAL277; electrostatic interactions included ARG53, LYS55, ASP275; And docking results showed MAF had a good binding capacity with Asp 275 [Figure 1A]. The docking further showed the binding between Asp275 and MAF still remained a structural cavity, and the binding position was mainly -NH2 of sulfonamide. The aminomethyl group at the para position of the benzene ring was mainly positively ionized under the action of H^+^, which had little contribution to small molecule binding [Figure 1B]. In view of the docking results, we planned to add a group to the amino position of the sulfonamide from the perspective of skeleton migration to fill the cavity through hydrogen bonding. And the aminomethyl group at the para position of the benzene ring was replaced by -CN, which can increase the compound solubility and improve the fat solubility. Secondly, the binding of π-π could be enhanced through the unsaturated bond of CN, which can improve the binding between small molecules and proteins [Figure 1C-1D] and [Figure 2A-2B]. For the synthesis of sulfonamide compound, benzonitrile was mainly used as the initial compound, which further underwent acylation reaction with chlorosulfonic acid under Fe catalysis. Toluene was used as solvent, benzonitrile: chlorosulfonic acid: Fe (1:1.1:0.1) was synthesized, with thin layer chromatography to detect the reaction progression, and 4-cyanobenzenesulfonic acid was finally hydrolyzed and formed under H_2_O catalysis. In the chlorination reaction, DMF:BTC (3:1) could form Vilsmeier reagent at 0^o^C and undergo chlorination with 4-cyanobenzenesulfonic acid at 0-60^o^C, followed by reduced pressure distillation after the reaction was completed (c). To be specific, three folds of triethylamine was used as a catalyst to synthesize sulfonamide, followed by purification by recrystallization or column chromatography [Figure 2C].


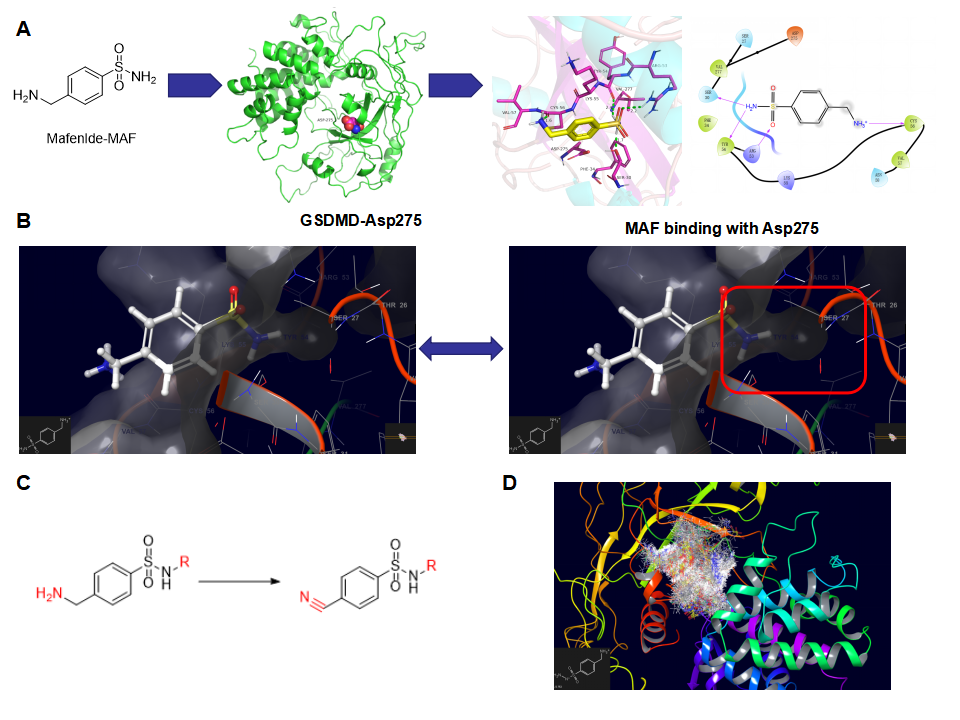


Figure 1: The virtual docking between sulfonamide small molecules (lead compounds) and GSDMD-Asp275, and the design principle of small molecule

A: Docking results between mafenide (the lead compound) and GSDMD-Asp275: hydrogen bonds: SER30, ARG53, TYR54, CYS56; hydrophobic interactions: PHE34, TYR54, CYS56, VAL57, VAL277; electrostatic interactions: ARG53, LYS55, ASP275.

B: For the binding mode between mafenide and Asp275, there was a cavity in GSDMD-Asp275, and the 4-aminomethyl group formed a hydrogen bond to stabilize the binding of small molecules. The cavity could be filled by sulfonamide modification to improve the stability of the binding.

C-D: The design principle of small molecule: The amino position of sulfonamide was further added with groups to fill the cavity through hydrogen bonding. The aminomethyl group at the para position of the benzene ring was replaced by -CN, which could increase the solubility of the compound and improve fat solubility. Moreover, the binding of π-π could be enhanced through the unsaturated bond of CN, which further improved the binding between small molecules and proteins.


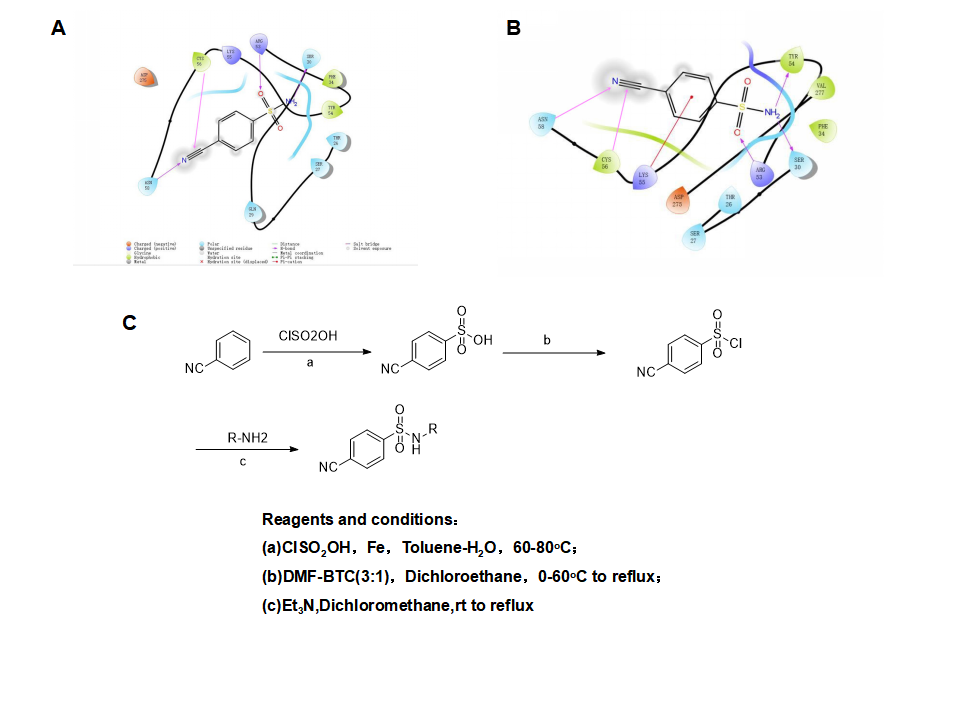


Figure 2: Docking results of lead small molecule and the synthesis method of sulfonamide small molecules

A: The binding mode of 4-cyanobenzenesulfonamide and GSDMD-Asp275. The -CN modification could improve binding stability by further binding to the protein through unsaturated bonds.

B: Synthesis method of sulfonamide small molecule compounds.
